# Supplementary material for: Benchmarking challenging small variants with linked and long reads
Source: Cell Genom. Author manuscript; Available in PMC 2022 Nov 29. (PMC9706577; doi:10.1016/j.xgen.2022.100128)
Supplement: Supplementary file 1 [file NIHMS1809018-supplement-1.pdf]

Cell Genomics, Volume *2*

Supplemental information

Benchmarking challenging small variants

with linked and long reads

Justin Wagner, Nathan D. Olson, Lindsay Harris, Ziad Khan, Jesse Farek, Medhat Mahmoud, Ana Stankovic, Vladimir Kovacevic, Byunggil Yoo, Neil Miller, Jeffrey A. Rosenfeld, Bohan Ni, Samantha Zarate, Melanie Kirsche, Sergey Aganezov, Michael C. Schatz, Giuseppe Narzisi, Marta Byrska-Bishop, Wayne Clarke, Uday S. Evani, Charles Markello, Kishwar Shafin, Xin Zhou, Arend Sidow, Vikas Bansal, Peter Ebert, Tobias Marschall, Peter Lansdorp, Vincent Hanlon, Carl-Adam Mattsson, Alvaro Martinez Barrio, Ian T. Fiddes, Chunlin Xiao, Arkarachai Fungtammasan, Chen-Shan Chin, Aaron M. Wenger, William J. Rowell, Fritz J. Sedlazeck, Andrew Carroll, Marc Salit, and Justin M. Zook

Supplementary Information:
Benchmarking challenging small variants with linked and long reads

Table S1. **Coverage of each sample by linked-reads and long-reads, related to STAR Methods**. PacBio HiFi calculated from median depth in VCFs used in integration pipeline. 10x Genomics coverage estimates are from the sequencing provider. ONT coverage calculated from median of mosdepth 1000 bp windows in bam file (note that variants from ONT were not used in v4.2.1; it was only used to exclude regions with abnormal coverage).

	Reference	PacBio HiFi coverage	10x coverage	ONT coverage
HG001	GRCh37	68	75	37
HG001	GRCh38	67	75	37
HG002	GRCh37	54	84	59
HG002	GRCh38	54	84	59
HG003	GRCh37	62	71	84
HG003	GRCh38	63	71	85
HG004	GRCh37	60	69	85
HG004	GRCh38	60	69	85
HG005	GRCh37	47	53	57
HG005	GRCh38	47	53	59
HG006	GRCh37	67	53	51
HG006	GRCh38	67	53	51
HG007	GRCh37	56	53	41
HG007	GRCh38	56	53	41

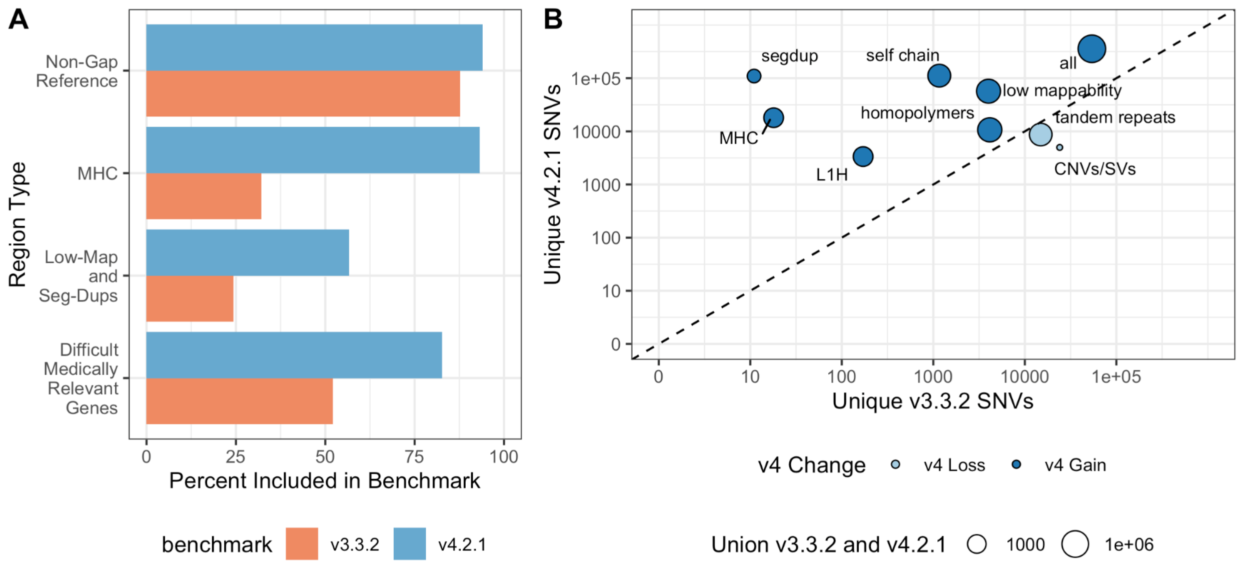

Figure S1. **New benchmark set includes more of the reference genome and more SNVs and indels, related to Figure 1.** (A) Percent of the genomic region that is included by v3.3.2 and v4.2.1 of all non-gap, autosomal GRCh37 bases; MHC; low mappability regions and segmental duplications; and 159 difficult-to-map, medically-relevant genes described previously. (B) The number of unique SNVs by genomic context. Circle size indicates the total number of SNVs in the union of v3.3.2 and v4.2.1. Circles above the diagonal indicate there is a net gain of SNVs in the newer benchmark, and circles below the diagonal indicate a net loss of SNVs in the newer benchmark.**.**

Table S3. Errors previously identified in v3.3.2 now corrected in v4.2.1, related to Table 1. Errors in v3.3.2 identified by PacBio Hifi that are corrected in v4.2.1, either matching PacBio HiFi callset or removed from benchmark regions.

Chromosome	Position	Result	Region Type
4	11,468,804	Outside v4.2.1 benchmark regions	
5	42,740,225	Outside v4.2.1 benchmark regions	LINE:L1PA2
2	5,143,996	Call matches in benchmark region	
13	48,291,499	Outside v4.2.1 benchmark regions	LINE:L1PA3
8	5,930,728	Outside v4.2.1 benchmark regions	
15	41,943,823	Outside v4.2.1 benchmark regions	
6	9,737,425	Outside v4.2.1 benchmark regions	
7	157,385,671	Reference call in benchmark regions	
17	32,064,214	Outside v4.2.1 benchmark regions	
1	94,256,825	Call matches in benchmark region	LINE:L1PA2
2	153,864,971	Call matches in benchmark region	LINE:L1HS
4	112,819,087	Call matches in benchmark region	LINE:L1HS
4	165,026,074	Call matches in benchmark region	LINE:L1PA2
11	23,338,682	Call matches in benchmark region	LINE:L1P1
1	35,034,071	Call matches in benchmark region	LINE:L1HS
3	79,181,734	Call matches in benchmark region	LINE:L1HS
4	94,532,444	Call matches in benchmark region	LINE:L1HS
8	46,873,565	Outside v4.2.1 benchmark regions	
9	22,350,168	Call matches in benchmark region	LINE:L1PA2
21	42,288,851	Call matches in benchmark region	LINE:L1PA2

Table S5. Expanded inclusion of difficult, medically relevant genes, related to Figure 4. Benchmark inclusion of 159 medically relevant genes totaling 10,009,480 bp in GRCh38 and 10,152,047 bp in GRCh37 on chromosomes 1-22 previously identified as difficult for short reads. bp included is the total number of bp included by each benchmark set and percent of bases included from the gene set.

Benchmark Set	Reference	bp included	SNVs	INDELS
v3.3.2	GRCh38	5,362,837 (54%)	6,242	943
v4.2.1	GRCh38	8,786,005 (88%)	10,175	1,469
v3.3.2	GRCh37	5,283,743 (52%)	6,364	997
v4.2.1	GRCh37	8,428,864 (83%)	10,710	1,471

Table S7. Curation of potential errors identified by Platinum Genomes, related to Figure 5: Manual curation results of 10 random sites in HG002 v4.2.1 that match Category 1 SNVs in Platinum Genomes.

Chromosome	Position	Curation	Notes
16	18288432	Selfchain/segdup	Many variants on one HP CCS, in selfchain/segdup
19	54726776	Selfchain/segdup	Cluster of variants in 10x/Illumina nearby and CCS has more variants on one HP than the other. In high depth selfchain/segdup that is smaller than 10kb
19	41379908	Selfchain/segdup	Cluster of variants, in LINE:L1MA3. In depth 2 segdup and depth 2 selfchain
8	11872949	Selfchain/segdup	Potential SV in segdup, since CCS and ONT have clipped reads nearby. Cluster of CAT1 variants
15	20360478	Possible CNV	Likely CNV given CCS data and high coverage in ONT. Several CAT1 variants in the region.
8	7223157	Selfchain/segdup	Cluster of CAT1 variants, in segdup and normal coverage but in a cluster of variants on one HP CCS and ONT, so may be more complex
15	20453992	Possible CNV	Cluster of CAT1 variants, cluster of variants on one HP CCS, and large change in coverage in CCS and nearby SV
7	149749666	Possible CNV	Cluster of CAT1 variants. Large changes in coverage in region in CCS data but overall looks reasonable.
15	20454464	Possible CNV	Many CAT1 variants in region, large change in CCS coverage in region, near what appears to be SV that is excluded from v4.2.1
12	74899879	Possible CNV	Somewhat elevated CCS coverage. In LINE:L1PA3.

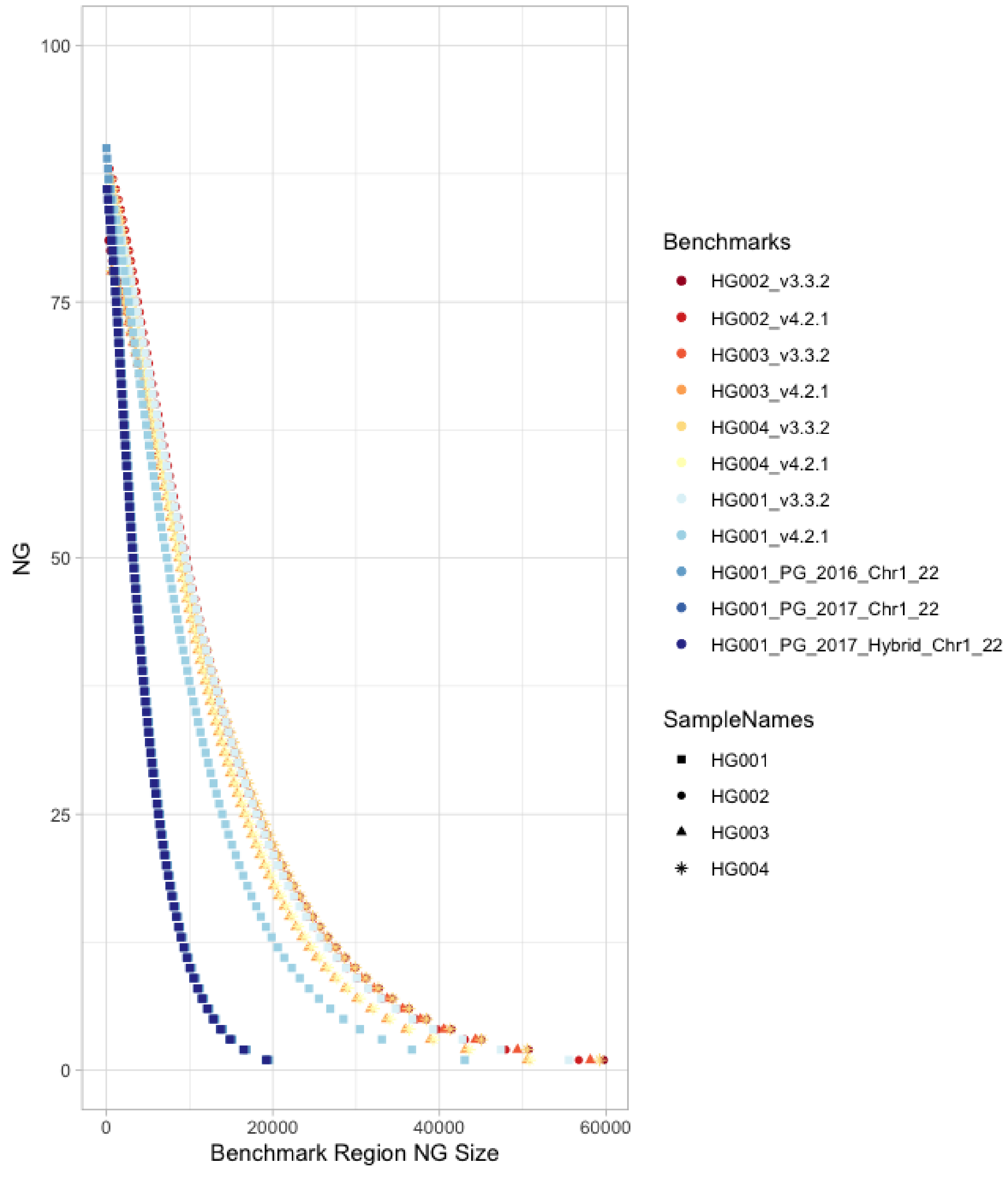

Figure S2. Comparison of benchmark region sizes between GIAB versions and Platinum Genomes, related to Figure 5. NG is the percent of the GRCh38 reference covered by benchmark regions at least as large as the Benchmark Region NG Size. This metric is analogous to Assembly NG50 except that benchmark region size is used in place of contig length. The contiguity of the benchmark improves in v4.2.1 compared to v3.3.2 and all versions of Platinum Genomes (PG).

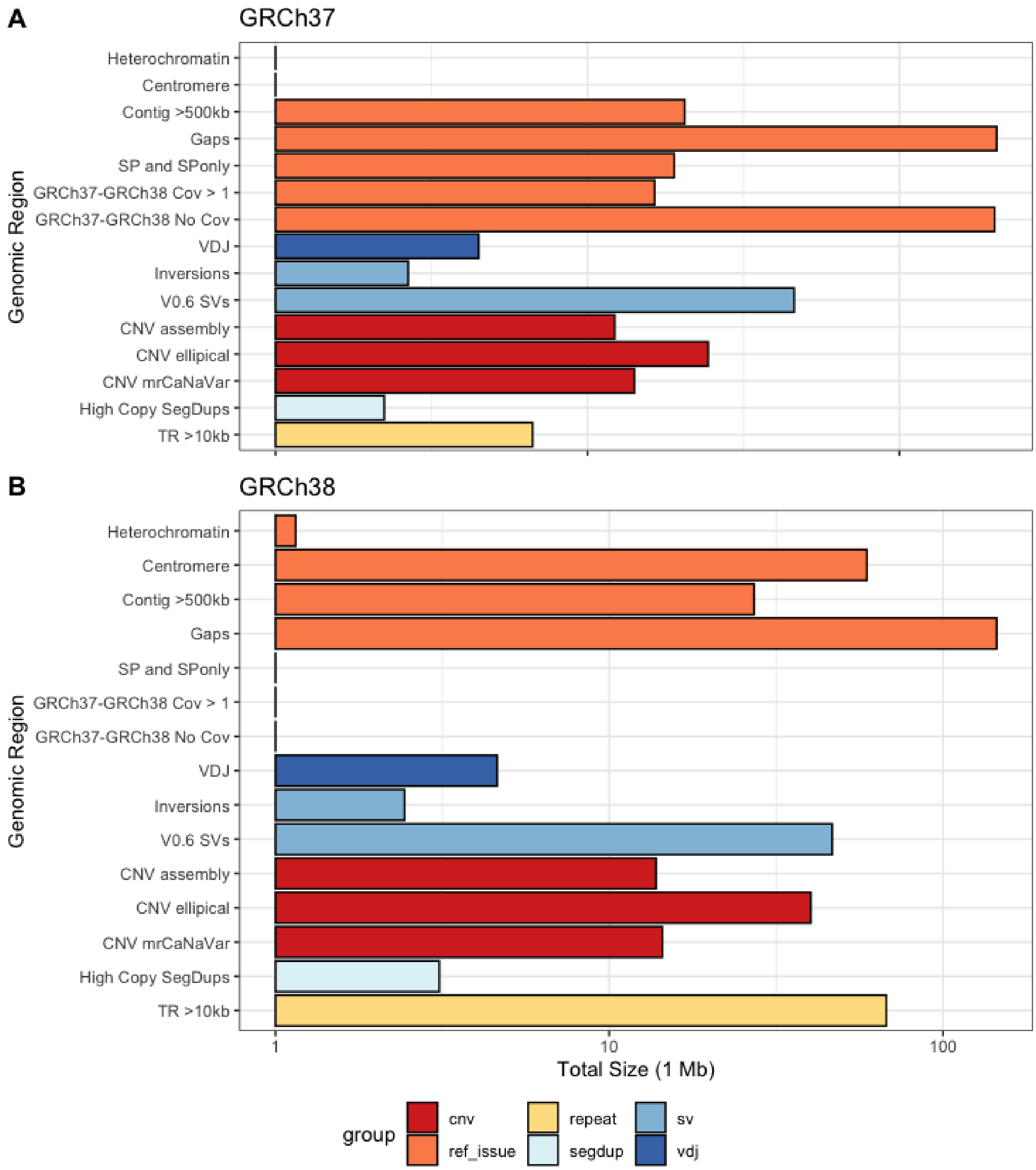

Figure S3. Base pairs in genomic regions excluded for all input variant call sets, related to Table 2.

Table S9. Problematic regions in GRCh38 v3.3.2 that were near or in the centromere, related to Table 2.

GIAB Sample	Chromosome	Start	End	Region Type
HG002	chr8	43637994	43672749	Centromere
HG002	chr8	43603010	43637285	Centromere
HG002	chr8	43831369	43864819	Centromere
HG002	chr7	62742402	62800702	q11.21 (near centromere)
HG002	chr7	57925899	57969199	p11.1 (centromere)
HG002	chr7	54317738	54350806	p11.2
HG002	chr7	62821943	62851720	q11.21
HG002	chr5	46337535	46371375	Centromere
HG002	chr5	46009909	46041150	Centromere
HG002	chr3	90613721	90676762	Centromere
HG002	chr3	90268364	90303792	Centromere
HG002	chr3	90445745	90478995	Centromere
HG002	chr19	27523978	27570562	Centromere
HG002	chr12	37624574	37664823	Centromere
HG002	chr12	34536432	34575253	Centromere
HG002	chr12	34483102	34520344	Centromere
HG002	chr12	37342974	37379851	Centromere
HG002	chr11	50785848	50821348	p11.12 (near centromere)
HG002	chr10	39052350	39083950	Centromere
HG002	chr10	39116363	39147923	Centromere
HG003	chr8	43601909	43637285	Centromere
HG003	chr8	43637994	43672749	Centromere
HG003	chr7	62742945	62800702	q11.21 (near centromere)
HG003	chr5	50193424	50229094	Centromere
HG003	chr5	46337535	46371375	Centromere
HG003	chr4	8843663	8892454	p16.1
HG003	chr3	90598264	90676761	Centromere
HG003	chr3	90268364	90303792	Centromere
HG003	chr3	90411794	90445037	Centromere
HG003	chr3	90445745	90478939	Centromere
HG003	chr22	22145576	22178716	q11.22

HG003	chr19	27523978	27577850	Centromere
HG003	chr12	37624574	37664823	Centromere
HG003	chr12	34536383	34575253	Centromere
HG003	chr12	34482540	34520344	Centromere
HG003	chr12	37342974	37379851	Centromere
HG003	chr11	50772422	50821348	p11.12 (near centromere)
HG003	chr10	39013337	39083750	Centromere
HG003	chr10	39116363	39153579	Centromere
HG003	chr10	39183589	39216647	Centromere
HG004	chr8	43637994	43672749	Centromere
HG004	chr8	43831342	43864819	Centromere
HG004	chr7	62742945	62815024	q11.21
HG004	chr7	57925899	57969199	Centromere
HG004	chr5	46009909	46041150	Centromere
HG004	chr5	50193424	50223736	Centromere
HG004	chr4	144161988	144192833	q31.21
HG004	chr3	90445745	90478957	Centromere
HG004	chr3	90507640	90536550	Centromere
HG004	chr2	88861923	88891174	p11.2
HG004	chr19	27523978	27559503	Centromere
HG004	chr12	37263197	37300537	Centromere
HG004	chr12	37342828	37379552	Centromere
HG004	chr12	63767401	63796912	q14.2
HG004	chr12	37815851	37844396	Centromere
HG004	chr12	34492116	34520344	Centromere
HG004	chr11	50772422	50806467	p11.12
HG004	chr10	39120199	39153579	Centromere
HG004	chr10	39055460	39087970	Centromere
HG004	chr10	39183589	39213934	Centromere

Table S10. Changes made to the benchmark formation process from v4alpha to v4.2.1, related to STAR Methods. The Genome in a Bottle Consortium has an iterative evaluation process to ensure new benchmarks are useful for assessing performance across diverse sequencing technologies and variant calling methods. The first version using PacBio HiFi and 10x Genomics data was v4alpha. In particular, GIAB found that some regions contained unreliable calls across technologies, and these regions were excluded from subsequent releases. In addition, the PacBio HiFi data changed during releases as new data were collected, and the 10x Genomics data remained constant at ~84x coverage.

**The v4alpha release** used PacBio Sequel I HiFi ~15 kb reads at ~28x coverage, with the difficult regions below (Bold entries changed from v3.3.2):	
**Difficult Region Description**	**Method Excluded From**
All candidate structural variant regions from the Son-Mother-Father Trio	All methods
All tandem repeats < 51bp in length	All methods except GATK from Illumina PCR-free, Complete Genomics, and **PacBio CCS DeepVariant**
All tandem repeats > 51bp and < 200bp in length	All methods except GATK from Illumina PCR-free and **PacBio CCS DeepVariant**
All tandem repeats > 200bp in length	All methods except **PacBio CCS DeepVariant**
Perfect or imperfect homopolymers > 10bp	All methods except GATK from Illumina PCR-free
Segmental duplications from Eichler *et al.*	All methods except **10X Genomics and PacBio CCS**
Segmental duplications > 10Kbp from self-chain mapping	All methods except **10X Genomics and PacBio CCS**
Regions homologous to contigs in hs37d5 decoy	All methods except **10X Genomics and PacBio CCS**
Difficult to map regions for short reads	All methods except **10X Genomics and PacBio CCS**
Homopolymer > 6bp in length	**All methods except GATK from Illumina PCR-free and Complete Genomics**
**The v4beta release** used PacBio Sequel I HiFi ~15 kb reads at ~28x coverage. Additionally, v4beta used additional tandem repeat files from UCSC, excluded the entire tandem repeat if any part was not in the benchmark BED, and changed the difficult regions below:	
**Difficult Region Description**	**Method Excluded From**
v0.6 SV Benchmark	All methods
Regions that are collapsed and expanded from GRCh37/38 Primary Assembly Alignments (corrected)	All methods
Diploid assemblies exhibit more than 2 contigs aligned > 10kb	All methods
Intersected short and long read based copy number > 2.5 (updated)	All Methods

Segmental duplications > 10Kb, Identity > 99%, Count > 5	All methods
mrCaNaVar duplications > 10kb (052119)	All methods except 10X Genomics and PacBio CCS
Outliers from long read coverage	All Methods
LINE:L1Hs > 500	All methods except Illumina MatePair, 10X Genomics, and PacBio CCS
All Tandem Repeats > 10kb in length	All methods

The v4.0 release used PacBio Sequel II HiFi ~11 kb reads at ~32x coverage, updated to DeepVariant v0.8, and kept the same exclusion regions as v4beta

The v4.1 release used PacBio Sequel II HiFi ~15 kb and ~20 kb reads at ~52x coverage. The diploid assembly-based MHC benchmark was used for the MHC region in v4.1. We also added the difficult regions below:

Difficult Region Description	Method Excluded From
**Potential copy number variation including CCS and ONT outlier and CCS, ONT, mrCanavar intersection**	**All methods**
**VDJ**	**All methods**
**Inversions**	**All methods**

The v4.2 release is the first for HG003 and HG004, and it used hifiasm to perform the assembly of PacBio HiFi reads in the MHC, and used dipcall with this assembly to call variants, including in segmental duplications that were previously not assembled properly. Since it represents complex variants as individual SNVs and indels, dipcall helps improve partial credit in some cases for variants that are only partially called correctly by the query callset. We also excluded entire homopolymers and tandem repeats in the MHC if they were not completely covered by the benchmark bed. Since calls were made for HG003 and HG004 in addition to HG002, we also performed a trio Mendelian analysis and excluded Mendelian violations from the benchmark regions for all individuals (except putative de novo variants in HG002 were not excluded from the benchmark regions).

The v4.2.1 release is for HG002, HG003, and HG004 on both GRCh37 and GRCh38. We now use the same MHC hifiasm approach for HG002 as with HG003 and HG004. We exclude SVs from a pbsv call set from HG003 and HG004 in addition to the GIAB v0.6 SV benchmark. A final update is that we exclude the KIR region because of highly variable copy number.

Table S11. Sequencing technology, mapping or assembler method, and variant caller that was used to generate each evaluation call set, related to Figure 5. The names used in **Figure 5** are in the fourth column.

**Sequencing Technology**	**Variant Caller**	**Mapper/Assembler**	**Figure 5 Name**
PacBio HiFi	DeepVariant	minimap2	PB DV-mm2
PacBio HiFi	GATK4	minimap2	PB GATK4-mm2
PacBio HiFi	Clair	minimap2	PB Clair-mm2
PacBio HiFi	DV	Duplomap	PB DV-Duplomap
PacBio HiFi	dipcall	WHDenovo	PB Dipcall-WHDenovo
Illumina PCR-Free TruSeq 2x250bp	Dragen	Dragen	Ill Dragen
Illumina PCR-Free TruSeq 2x250bp	Dragen	VG	Ill Dragen-VG
Illumina PCR-Free HiSeq 2x150bp	SevenBridges	SevenBridges Graph Aligner	Ill SevenBridges GRAF
Illumina PCR-Free HiSeq 2x150bp	xAtlas	NovoAlign	Ill xAtlas
Illumina PCR-Free NovaSeq 2x250bp	GATK	BWA	Ill GATK-BWA
10x Genomics	LongRanger	LongRanger	10x LongRanger
10x Genomics	paftools	Aquila	10x paftools-Aquila
ONT	Clair	minimap2	ONT Clair-mm2
ONT	Clair	NGMLR	ONT Clair-ngmlr

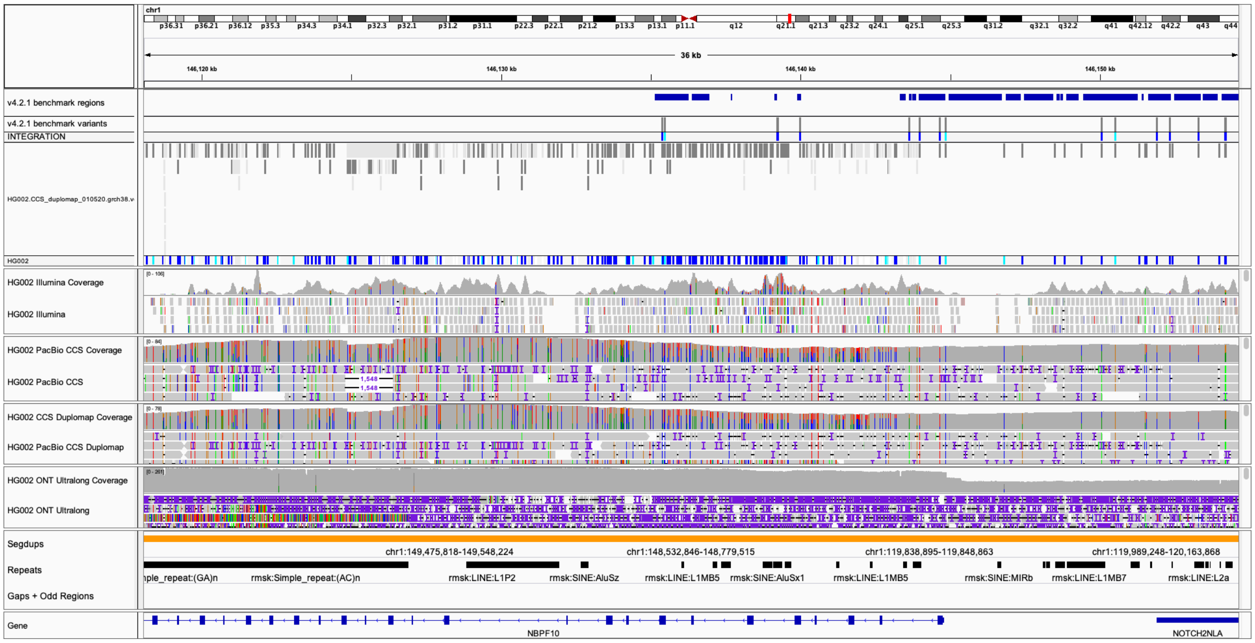

Figure S4. Error in benchmark due to large duplication in HG002, related to Figure 5. The benchmark and callset both make calls in this region where there is likely a large duplication in HG002 compared to GRCh38 that was not detected by our exclusion criteria. This specific example is a FP SNP in PacBio HiFi Duplomap DV where the benchmark region indicates a reference call at this location.

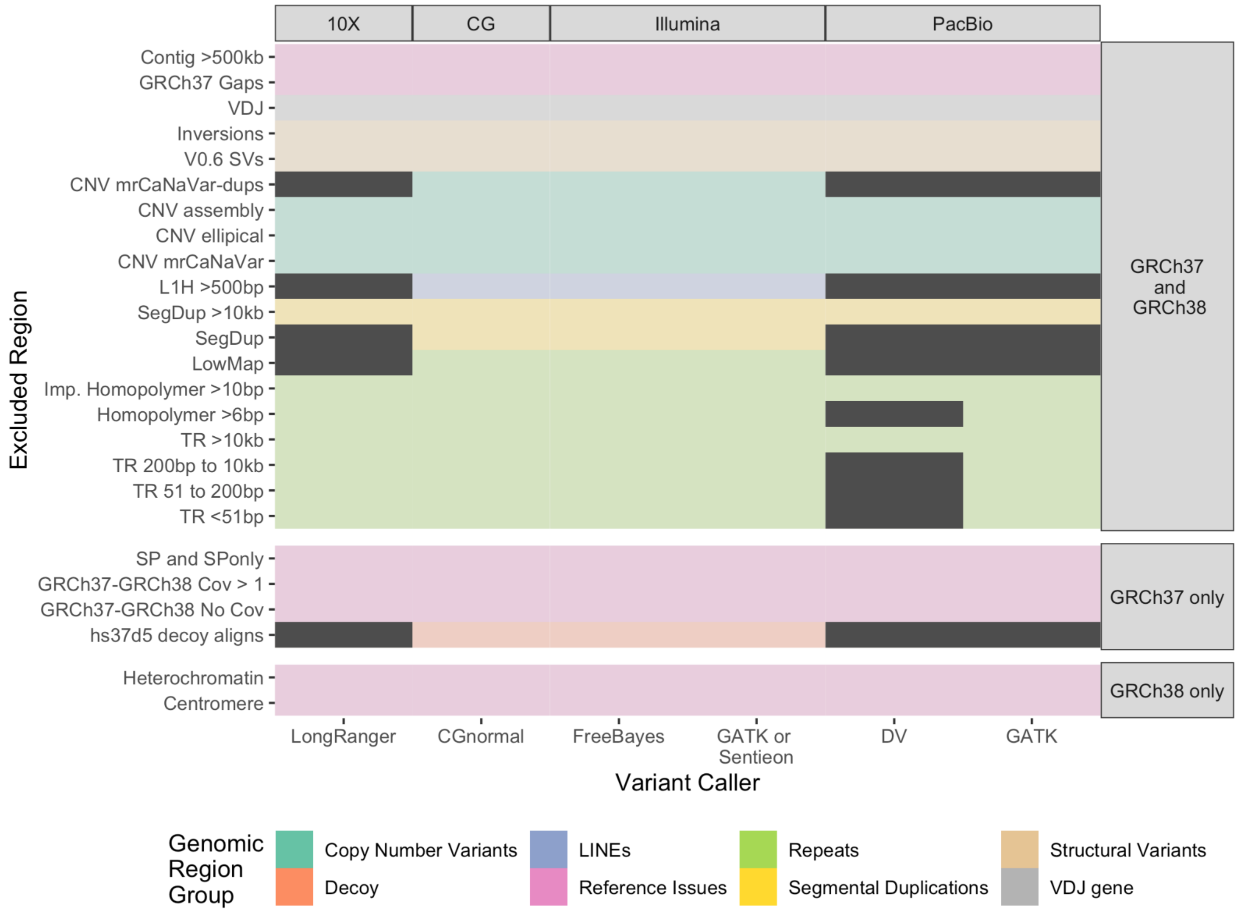

Figure S5: Genomic regions included by input variant callset, related to STAR Methods. Genomic regions are excluded based on the biases of each technology that decrease reliability of variants in particular regions. Included regions are indicated by dark grey. Illumina PCR-Free includes both the high coverage HiSeq 300x and 2x250 HiSeq datasets. The PacBio HiFi dataset consists of 4 SMRT Cells of 15 kb libraries and 2 SMRT Cells of 20 kb libraries.

Table S14. Long Range PCR Components, related to STAR Methods

	LINEs	*C4A*	*C4B*	*Cyp21A2*	*Cyp2D6*	*DMBT1*	*HSPG2*	*PMS2*	*STRC*	*TnxA*	*TnxB*
Buffer (5X)	1X	1X	1X	1X	1X	1X	1X	1X	1X	1X	1X
dNTP (250uM each)	250uM	400uM	400uM	250uM	0.3mM	400uM	200uM	400uM	400uM	250uM	250uM
Forward Primer	0.25uM	0.5uM	0.5uM	10uM	0.5uM	0.4uM	0.3uM	0.2uM	0.4uM	10uM	10uM
Reverse Primer	0.25uM	0.5uM	0.5uM	10uM	0.5uM	0.4uM	0.3uM	0.2uM	0.4uM	10uM	10uM
Polymerase (1.25 units/uL)	1.25 U	1.25 U	1.25 U	1.25 U	1.25 U	2.5 U	0.5 U	1.25 U	2 U	0.5 U	0.5 U
DNA	300ng	100ng	100ng	250ng	1uL	2uL	300ng	100ng	300ng	250ng	250ng
Water	To 50uL	To 50uL	To 50uL	To 30uL	To 25uL	To 50uL	To 50uL	To 25uL	To 50uL	To 30uL	To 30uL

Table S15. Long Range PCR Conditions, related to STAR Methods

Gene	PCR Conditions
LINEs	30 cycles of 98°C for 10 seconds, 60°C for 15 seconds, and 68°C for 8 minutes.
C4A	98°C for 2 minutes; followed by 40 cycles of 98°C for 45 seconds, 66°C for 60 seconds, and 72°C for 9 minutes, with a final extension step of 72°C for 10 minutes.
C4B	98°C for 2 minutes; followed by 8 cycles of 94°C for 45 seconds, 64°C for 60 seconds, with a decrease of 0.5°C per cycle, and 72°C for 9 minutes; followed by 30 cycles of 94°C for 45 seconds, 59°C for 60 seconds, and 72°C for 9 minutes, with an increase of 10 seconds per cycle, with a final extension step of 72°C for 15 minutes
Cyp21A2<br>*TnxA*<br>*TnxB*	94°C for 4 minutes; followed by 12 cycles of 94°C for 30 seconds, 62°C for 40 seconds, and 68°C for 5 minutes; followed by 16 cycles of 94°C for 30 seconds, and 68°C for 5 minutes.
Cyp2D6	96°C for 30 seconds; followed by 30 cycles of 94°C for 15 seconds, 68°C for 30 seconds, and 68°C for 7 minutes, with a final extension step of 68°C for 30 minutes.
DMBT1	94°C for 1 minute; followed by 30 cycles of 98°C for 10 seconds, and 68°C for 15 minutes, with a final extension step of 72°C for 10 minutes.
HSPG2	30 cycles of 98°C for 10 seconds, 60°C for 15 seconds, and 68°C for 10 minutes.
PMS2	94°C for 1 minute; followed by 35 cycles of 94°C for 10 seconds, and 65°C for 30 seconds, and 68°C for 15 minutes, with a final extension step of 72°C for 10 minutes.
STRC	93°C for 3 minutes; followed by 38 cycles of 93°C for 15 seconds, 64°C for 30 seconds, and 68°C for 17 minutes, with a final extension step of 68°C for 5 minutes.
